# Comparison of outcomes for patients with primary sclerosing cholangitis associated with ulcerative colitis and Crohn’s disease

**DOI:** 10.1093/gastro/gou074

**Published:** 2014-10-29

**Authors:** Udayakumar Navaneethan, Preethi GK Venkatesh, Ramprasad Jegadeesan, Vennisvasanth Lourdusamy, Jeffrey P Hammel, Ravi P Kiran, Bo Shen

**Affiliations:** ^1^Department of Gastroenterology, Digestive Disease Institute, The Cleveland Clinic, Cleveland, OH, USA; ^2^Department of Colorectal Surgery, Columbia University Medical Center, New York Presbyterian Hospital, New York, NY, USA.

**Keywords:** primary sclerosing cholangitis, ulcerative colitis, Crohn’s disease, colon neoplasia, colectomy, liver transplantation

## Abstract

**Background:** The comparative outcomes of ulcerative colitis (UC) and Crohn’s disease (CD) in patients with primary sclerosing cholangitis (PSC) are unclear; the aim of our study was to make an objective comparison.

**Methods:** A total of 273 patients with PSC and inflammatory bowel disease (223 with UC and 50 with CD) were included. Clinical and demographic variables were obtained.

**Results:** The PSC risk score was similar for both groups. The median follow-up period in patients with PSC-UC was 12 years (range 0–38) and that for PSC-CD was 14 years (range 1–36). The median number of disease flares per year was higher in PSC-UC patients than in the PSC-CD group [1*vs.*0 (ranges 0–20 and 0–9, respectively); *P < *0.001]. More patients with UC developed colon neoplasia than CD (35.9% *vs.*18%; *P = *0.009). On proportional hazards analysis for the risk of colectomy, UC patients had a 12% higher risk for colectomy [hazard ratio (HR) = 0.88; 95% confidence interval (CI) 0.51–1.51; *P = *0.64]. Liver transplantation for PSC was associated with decreased risk (HR = 0.57; 95% CI 0.37–0.89; *P = *0.013), while colon neoplasia increased the risk (HR = 3.83; 95% CI 2.63–5.58; *P <* 0.001) for colectomy. On proportional hazards analysis for the risk of colon neoplasia, UC patients had 56% higher risk of developing colon neoplasia than CD (HR = 0.44; 95% CI 0.16–1.25; *P = *0.12).

**Conclusions:** PSC patients with CD appear to be associated with a lower risk of colon neoplasia and colectomy than PSC patients with UC.

## Introduction

Primary sclerosing cholangitis (PSC) is commonly associated with underlying inflammatory bowel disease (IBD)—in particular ulcerative colitis (UC) [1, 2]. Crohn’s disease (CD) is less likely to be associated with PSC [3]. The clinical presentations and outcomes of UC and CD in the presence of PSC are unclear. Some authors argue that it would be difficult to differentiate UC from CD in the presence of PSC, as patients with a diagnosis of UC have rectal sparing and backwash ileitis that mimicks CD [4].

The risk of colon neoplasia with UC and CD has been studied and has been shown to be comparable [5, 6]. A population-based study from Canada suggested that the risk of colon neoplasia in patients with CD was similar to that of patients with UC [5]. In fact, absolute cumulative colon neoplasia frequencies for CD and UC have been shown to be nearly identical: 8% for UC and 7% for CD 20 years following diagnosis [6]. However very few patients with PSC were included in these studies and thus the impact of PSC on this risk cannot be clearly studied. Previous studies have shown that patients with PSC and UC are at significantly higher risk of developing colon neoplasia than UC patients without PSC [7–12]. In patients with CD and PSC, this evidence is less clear. In a study from Europe, colonic CD increased the risk of colon neoplasia [4]; however, in another study from England comparing CD patients with and without PSC, colonic CD did not seem to increase the risk of neoplasia of the colon [13].

We have published our results relating to the impact of PSC on outcomes in our cohort of 167 UC- and 41 CD patients who were followed up to 2011 [14–16]. We reported the inverse relationship between the activity of PSC and UC, and the impact of severity of PSC on the outcomes of CD, in terms of colon neoplasia and colectomy [14, 15]. In our cohort, we also studied the impact of liver transplantation for PSC, on the natural history of UC activity before and after liver transplantation [16].

In an earlier study from England, primary sclerosing cholangitis with Crohn’s disease (PSC-CD) less commonly progressed to cancer, liver transplantation, or death than primary sclerosing cholangitis with ulcerative colitis (PSC-UC) [17]. That study included only 32 patients with CD and the authors suggested that the small sample size limited the value of the observations. To our knowledge, no direct comparison has been made—in terms of the disease course, risk of colon neoplasia and colectomy—of the clinical outcomes of North American UC and CD patients with associated PSC. In the author’s practice, it appears that UC patients run a higher risk of neoplasia than of Crohn’s colitis and hence appear to face increased risk of surgery. The aim of our study was to evaluate and compare the disease phenotype, disease course, colon neoplasia and colectomy in UC and CD in patient cohorts with associated PSC.

## Patients and methods

### Patients

This historical cohort study was approved by the Cleveland Clinic Institutional Review Board. We have previously described the manner in which the database was set up and populated; this was a retrospective database study [14–16]. We included all patients older than 18 years, with PSC concurrent with UC or CD. Patients with indeterminate colitis and patients with UC or CD, who did not have follow-up at the Cleveland Clinic to determine the natural history of their disease, were excluded. We used the standard definition of PSC based on imaging features at endoscopic retrograde cholangiopancreatography or magnetic resonance cholangiopancreatography [1].

A total of 273 patients suffering from primary sclerosing cholangitis concurrent with inflammatory bowel disease (PSC-IBD), who satisfied the selection criteria, were included in our cohort from 1985 to 2014. Of these, 50 had PSC-CD and 223 had PSC-UC.

### Data collection

Diagnoses of UC and CD were made, based on endoscopic examination, as well as on histological results [14–16, 18]. Patients with CD and PSC do not have the typical clinical features of CD [15], such as the presence of granulomas, pancolitis which is patchy on biopsy, the presence of small bowel involvement proximal to the ileum, the presence of fistulizing disease or small bowel strictures [19].

The following demographic and clinical variables were obtained from patient medical records:

- age
- gender
- smoking and alcohol history
- family history of IBD, PSC, or colon cancer in first degree relatives
- the year in which UC was diagnosed and its duration
- year in which PSC was diagnosed and its duration
- severity of PSC at diagnosis or earliest available calculated based on the Mayo PSC risk score [20]
- development of colon neoplasia and cholangiocarcinoma during the follow-up period
- outcomes (patient alive at last follow-up, dead or had liver transplantation)
- the use of long-term medical therapy including corticosteroids, immunomodulators including azathioprine/6-mercaptopurine, biologics and ursodeoxycholic acid.

We retrospectively defined the extent of UC and CD based on the Montreal Classification [20].

Disease activity in UC patients was assessed endoscopically using the Schroeder (Mayo Score) [22], and CD Index of Severity based on colonoscopy at diagnosis or earliest available at our institution [23]. In cases where this information was not available—particularly in patients who had undergone colonoscopy prior to 1998 and colonoscopy performed by non-IBD physicians, the endoscopist’s reported assessment was used. A disease flare of UC or CD was defined by the presence of clinical symptoms requiring a short course of corticosteroids. In our cohort, we collected information on flares during the final 5 years of follow-up.

Our study patients underwent colonoscopic surveillance for the development of colonic neoplasia or colon cancer every 1–2 years. Information on the presence of dysplasia was obtained from the pathology reports and dysplasia was considered to be flat if there was no documentation of a raised mass, lesion, or polyp in endoscopy or pathology reports.

### Outcome measurement

The primary outcome of interest was the comparison of the phenotype and clinical outcome of UC and CD in PSC patients. We wanted specifically to compare the risk of disease flares and disease course, development of colon neoplasia, and the risk of colectomy in PSC patients with UC and CD.

### Statistical analysis

Descriptive statistics were computed for all factors. These include medians, 25th and 75th percentiles, range, or mean and standard deviation for continuous factors, and frequencies and percentages for categorical factors. We used Wilcoxon’s rank sum tests for continuous factors and Pearson’s chi-squared or Fisher’s exact tests for categorical factors.

Survival rates were estimated using a Kaplan-Meier approach from the date of diagnosis of PSC to the date of death or liver transplantation. Patients who were alive at the time of last follow-up were censored. The Kaplan-Meier curve for patient’s survival in patients with PSC-UC and PSC-CD was compared using the log-rank test.

A Cox logistic regression model was constructed by including variables that had significant univariate associations with colectomy and colon neoplasia, and then performing backward stepwise selection with a removal criterion of *P > *0.05. All analyses were performed using R 2.10.1 software (The R Foundation for Statistical Computing, Vienna, Austria).

## Results

### Demographic characteristics

The basic demographic and clinical information, including age, sex, age at PSC diagnosis and colonoscopic extent of UC or CD are summarized in Table 1. The male-to-female ratio was slightly lower in patients with PSC-CD, although the difference was not statistically significant. The age at diagnosis of PSC and age of diagnosis of UC or CD was also similar in each group.

**Table 1.** Comparison of demographic and clinical variables between primary sclerosing cholangitis patients with ulcerative colitis and Crohn’s disease (for liver-related outcomes)

Variable	PSC with CD	PSC with UC	*P*-value
	*n* = 50	*n* = 223	
Age at diagnosis of PSC (years, mean ± SD)	37.8 ± 13.1	38.3 ± 13.1	0.73
Male (*n*, %)	30 (60.0%)	153 (68.6%)	0.24
Body mass index [g/m^2^, median (interquartile range)]	26.4 (22.1–28.8)	25.0 (22.3–28.1)	0.56
Smoker (*n*, %)			0.02
Yes	6 (12.0%)	8 (3.6%)	
Ex-smoker	6 (12.0%)	21 (9.4%)	
Alcohol (*n*, %)			0.008
Yes	8 (16.0%)	27 (12.1%)	
Ex-alcohol use	2 (4%)	5 (2.2%)	
Bile duct involvement (*n*, %)			0.08
Intrahepatic only	8 (16%)	48 (21.5%)	
Extrahepatic only	10 (20%)	27 (12.1%)	
Intra-and extrahepatic	32 (64%)	148 (66.4%)	
Initial albumin [mg/dL, median (range)]	3.5 (0–5)	3.6 (1.6–5.2)	0.73
Initial bilirubin [mg/dL, median (range)]	1.4 (0–11.2)	2 (0.2–25.8)	0.47
Initial INR [median (range)]	1.0 (0–2.3)	1.0 (0.7–5.2)	0.11
Initial PSC Mayo risk score [median (range)]	1.06 (-2–3.43)	0.97 (-2.33–5.19)	0.41
Initial AST [IU/L, median (range)]	58 (0–278)	59 (9–691)	0.42
OLT during follow-up (*n*, %)	22 (44.0%)	99 (44.4%)	0.96
Age at OLT (years, median)	42	46	0.11

PSC = primary sclerosing cholangitis; UC = ulcerative colitis; CD = Crohn’s disease; OLT = orthotropic liver transplantation; SD = standard deviation; INR = international normalized ratio; AST = aspartate aminotransferase; OLT = orthotropic liver transplantation

### Clinical characteristics of primary sclerosing cholangitis

Patients in the PSC-UC group had similar serum bilirubin, aspartate aminotransferase and serum albumin to patients in the PSC-CD group at the time of PSC diagnosis. Their median initial Mayo PSC risk score was also similar. The median follow-up period in patients with PSC-UC was 12 years (range 0–38), and in patients with PSC-CD was 14 years (range 1–36) (Table 1). No patient developed dysplasia or cancer before a diagnosis of PSC was made. Two patients underwent colectomy prior to PSC diagnosis.

### Disease distribution of inflammatory bowel disease

All patients with CD had colon involvement, while 10 patients had additional small bowel involvement. Patients with CD were classified as L2 (*n = *36), L2–L4 (*n = *4) and L3 (*n = *10) based on the Montreal Classification. Among the 40 patients with isolated colon involvement, extensive colitis was seen in 36, while the remainder had patchy colitis; granulomas were seen in 25 patients, fistulas in 3, while patchy colitis with deep ulcers was seen in the remaining 12. Among patients with PSC-UC, extensive colitis (E3) was seen in 207 individuals (92.8%), while left-sided colitis (E2) was seen in the remainder, based on the Montreal Classification. The median number of flares was higher in PSC-UC than in the PSC-CD group [1 *vs.*0 (ranges 0–20 and 0–9, respectively); *P < *0.001]. Table 2 summarizes the IBD activity for the PSC-UC and PSC-CD groups and the use of medications for UC and CD.

**Table 2.** Comparison of demographic and clinical variables between patients with primary sclerosing cholangitis patients with ulcerative colitis and Crohn’s disease (for colon-related outcomes)

Variable	PSC with CD	PSC with UC	*P*-value
	*n* = 50	*n* = 223	
Age at diagnosis of UC or CD (years, mean ± SD)	30.36 ± 13.84	32.09 ± 13.71	0.34
Duration of UC or CD prior to PSC diagnosis (years, mean ± SD)	8.64 ± 9.99	6.78 ± 8.85	0.40
Age at diagnosis of UC or CD and PSC (years, mean ± SD)	39.00 ± 13.80	38.84 ± 13.13	0.93
UDCA use (*n*, %)	38 (76.0%)	173 (77.6%)	0.81
UDCA dose [mg/d, median (range)]	900 (0–1500)	900 (0–3200)	0.17
Azathioprine/6-mercaptopurine use (*n*, %)	6 (12.0%)	20 (9.0%)	0.59
Biologics (*n*, %)	6 (12%)	13 (5.8%)	0.26
Corticosteroid use (*n*, %)	17 (41.5%)	106 (47.5%)	0.12
5-Aminosalicylate use (*n*, %)	30 (60.0%)	144 (64.6%)	0.16
Moderate/severe disease activity at diagnosis (*n*, %)	3 (7.3%)	28 (16.8%)	0.17
Number of UC or CD flares [median (range)]	0 (0–9)	1 (0–20)	<0.001
Colectomy during follow-up (*n*, %)	16 (32.0%)	107 (48.0%)	0.16
Age at colectomy [years, median (interquartile range)]	47 (32.8–55.3)	41 (30.5–48.0)	0.16
Dysplasia (*n*, %)			0.02
Low-grade	2 (4.0%)	17 (7.6%)	
High-grade	4 (8.0%)	29 (13.0%)	
Colon carcinoma (*n*, %)	3 (6.0%)	34 (25.6%)	0.03
Dysplasia and/or colon carcinoma (*n*, %)	9 (18.0%)	80 (35.9%)	0.009
Cholangiocarcinoma (*n*, %)	1 (2%)	9 (4.0%)	0.7

PSC = primary sclerosing cholangitis; UC = ulcerative colitis; CD = Crohn’s disease; UDCA = ursodexoxycholic acid; SD = standard deviation

### Colon neoplasia and cholangiocarcinoma

Nine of the 50 patients (18%) with PSC-CD developed colon neoplasia, as compared with 80/223 patients (35.9%) with PSC-UC (*P = *0.009). In the PSC-CD group, high-grade dysplasia was seen in four patients, low-grade dysplasia in two and colon cancer in three while, in the PSC-UC group, 17 had low-grade dysplasia, 29 had high-grade dysplasia and 34 had colorectal cancer. One patient (2.0%) in the PSC-CD group developed cholangiocarcinoma, compared with 9/223 patients (4%) in the PSC-UC group (*P = *0.7) (Table 2).

### Orthotropic liver transplantation

In the PSC-UC group, 99 (44.4%) of the 223 patients underwent orthotropic liver transplantation (OLT) on follow-up, compared with 22/50 (44%) in the PSC-CD group (*P = *0.96) (Table 1). The median age at the time of OLT was also similar in both groups (46 *vs.* 42 years, respectively; *P = *0.11), as was use of ursodexoxycholic acid (UDCA) (77.6% *vs.* 76.0%, respectively; *P = *0.81) and the median dose of UDCA (900 *vs.* 900 mL/d; *P = *0.17) (Table 2).

### Surgery for inflammatory bowel disease

One hundred and seven patients in the PSC-UC group (48.0%) underwent colectomy, in contrast to just 16 patients in the PSC-CD group (32.0%) (Table 2). Among these sixteen, nine underwent colectomy for steroid refractory/dependent disease, while the other seven underwent colectomy for dysplasia/colon cancer. Among the 107 patients in the PSC-UC group who underwent colectomy, 44 cases were for steroid refractory/dependent disease, while 61 were for dysplasia/colon cancer and, in the remaining two patients, for both steroid dependent disease and dysplasia.

Of these same 107 patients, 12 (11.2%) underwent total proctocolectomy with end ileostomy, while the remaining 95 had total proctocolectomy with ileal-pouch anal anastomosis (IPAA). Of the 16 patients who underwent colectomy for CD, 9 underwent total proctocolectomy with end ileostomy, while the remaining 7 underwent IPAA, as they did not have small bowel involvement.

### Survival analysis

**Figure 1.**
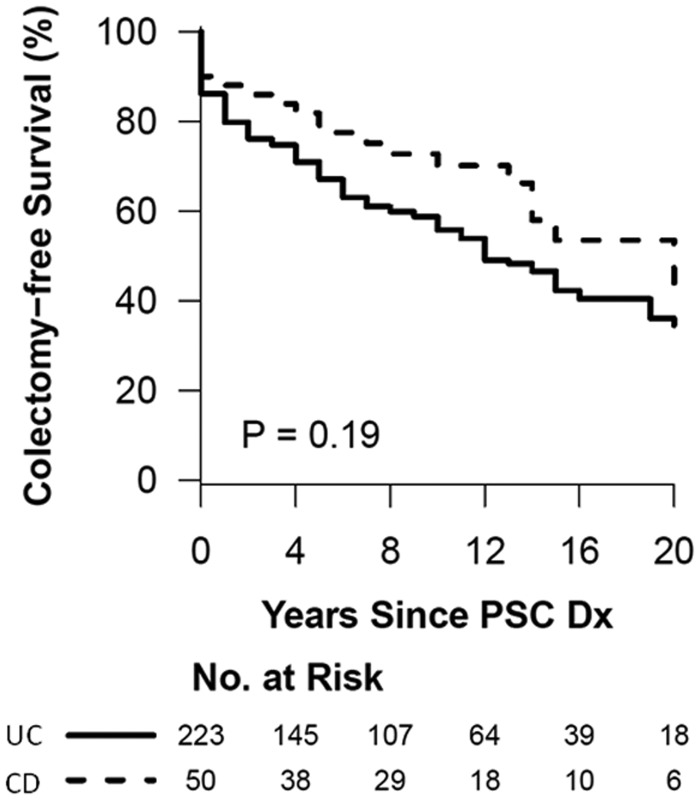
Kaplan-Meier survival curve for colectomy-free survival of primary sclerosing cholangitis patients with ulcerative colitis and Crohn’s disease

**Figure 2.**
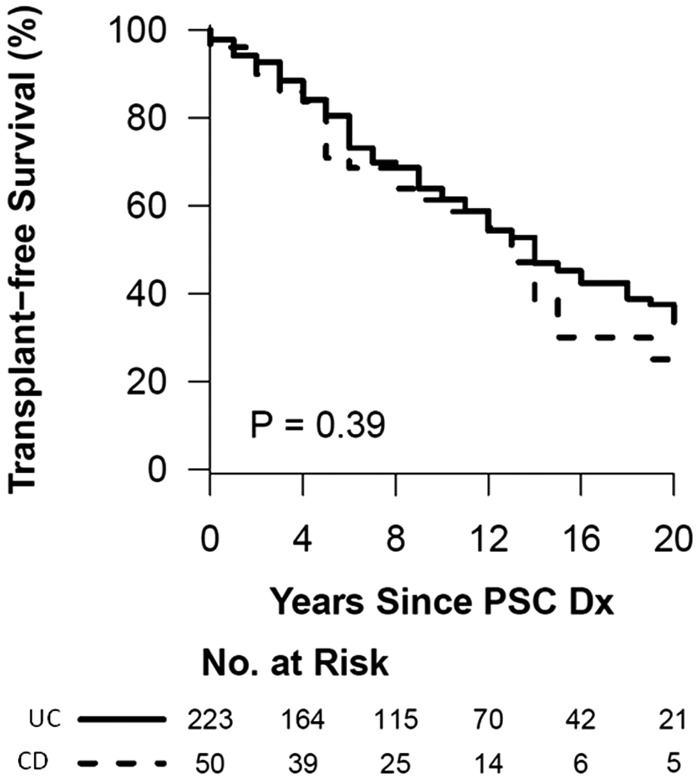
Kaplan-Meier survival curve for liver transplantation-free survival of primary sclerosing cholangitis patients with ulcerative colitis and Crohn’s disease

**Figure 3.**
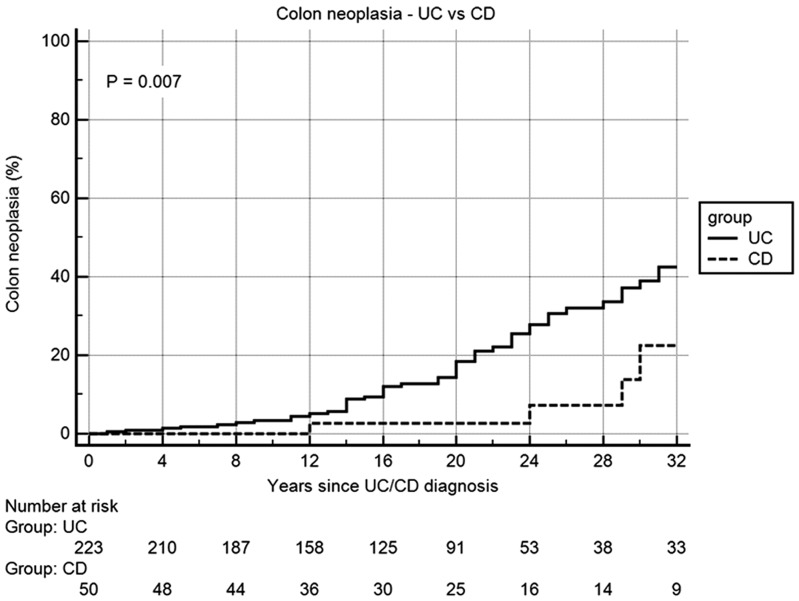
Kaplan-Meier survival curve for risk of colon neoplasia in primary sclerosing cholangitis patients with ulcerative colitis and Crohn’s disease

Figures 1 and 2 summarize the Kaplan-Meier curve of the survival analysis of patients in patients with either PSC-UC or PSC-CD. The colectomy-free survival and OLT-free survival was not significantly different between the groups. Figure 3 summarizes the risk of colon neoplasia in patients with either PSC-UC or PSC-CD. The risk of colon neoplasia was higher in patients with PSC-UC than in PSC-CD.

### Multivariate analysis for risk of colectomy

Table 3 summarizes the univariate risk factors for colectomy. On proportional hazards analysis, the presence of CD, as opposed to UC, was associated with a trend towards decreased risk [hazard ratio (HR) = 0.88; 95% confidence interval (CI) 0.51–1.51; *P = *0.64]. OLT for PSC (HR = 0.57; 95% CI 0.37–0.89; *P = *0.013) and a higher Mayo PSC risk score at diagnosis (HR = 0.53 95% CI 0.34–0.83; *P = *0.006) were associated with decreased risk of colectomy, while the development of colon neoplasia increased the risk (HR = 3.83; 95% CI 2.63–5.58; *P < *0.001) (Table 4).

**Table 3.** Univariate analysis of risk factors for colectomy

Variable	Odds ratio (95% CI)	*P*-value
Age	0.86 (0.6 − 1.23)	0.41
Male Gender	1.12 (0.77 − 1.62)	0.55
Serum albumin at diagnosis (>3.6 g/dL)	1.57 (1.09 − 2.25)	0.014
Serum bilirubin at diagnosis	0.41 (0.28 − 0.6)	<0.001
AST	0.64 (0.45 − 0.92)	0.015
Age of diagnosis of UC or CD	0.55 (0.38 − 0.79)	0.001
Duration of UC or CD prior to PSC diagnosis	2.92 (1.98 − 4.29)	<0.001
Age of diagnosis with PSC	0.89 (0.62 − 1.27)	0.52
UDCA use	1.07 (0.69 − 1.63)	0.77
Requirement for OLT	0.36 (0.24 − 0.53)	<0.001
Mayo PSC risk score	0.51 (0.35 − 0.73)	<0.001
5-Aminosalicylate use	1.15 (0.79 − 1.66)	0.47
Azathioprine/6-mercaptopurine use	1.99 (1.2 − 3.29)	0.006
Number of disease flares	4.37 (2.91 − 6.55)	<0.001
Presence of dysplasia/cancer	4.79 (3.33 − 6.88)	<0.001
Colon cancer	5.18 (3.21 − 8.36)	<0.001
CD (relative to UC)	0.6 (0.36 − 1.02)	0.06

AST = aspartate aminotransferase; UC = ulcerative colitis; CD = Crohn’s disease; PSC = primary sclerosing cholangitis; UDCA = ursodexoxycholic acid; OLT = orthotropic liver transplantation

**Table 4.** Cox proportional hazards analysis for colectomy

Variable	Hazard ratio (95% CI)	*P*-value
Duration of UC or CD prior to PSC diagnosis (per 5 years)	1.20 (1.08–1.32)	<0.001
Age of diagnosis with PSC (per 5 year)	1.01 (0.92–1.11)	0.80
Presence of dysplasia/cancer	3.83 (2.63–5.58)	<0.001
UDCA use	1.01 (0.65–1.55)	0.97
Requirement for OLT	0.57 (0.37–0.89)	0.013
Mayo PSC risk score = 1 (relative to 0)	0.53 (0.34–0.83)	0.006
CD (relative to UC)	0.88 (0.51–1.51)	0.64

CI = confidence interval; UC = ulcerative colitis; CD = Crohn’s disease; PSC = primary sclerosing cholangitis; UDCA = ursodexoxycholic acid; OLT = orthotropic liver transplantation

### Multivariate analysis for risk of colon neoplasia

Table 5 summarizes the univariate risk factors for colon neoplasia. On proportional hazards analysis, the presence of CD, when compared with UC, was associated with a trend towards decreased risk (HR = 0.44; 95% CI 0.16–1.25; *P = *0.12). Moderate-to-severe disease activity on endoscopy at the time of diagnosis (HR = 5.28; 95% CI 2.24–12.40; *P < *0.001) and duration of UC or CD (HR = 1.22; 95% CI 1.03–1.45; *P = *0.02) independently increased the risk of developing any colon neoplasia. Use of UDCA did not alter the risk of neoplasia in our cohort (HR = 1.31; 95% CI 0.62–2.78; *P = *0.48) (Table 6).

**Table 5.** Univariate analysis of risk factors for colon dysplasia/cancer

Variable	Odds ratio (95% CI)	*P*-value
Age	1.01 (0.99–1.04)	0.18
Male gender	1.56 (0.89–2.75)	0.12
Body mass index	0.95 (0.90–1.01)	0.08
Serum albumin at diagnosis	1.33 (0.91–1.95)	0.14
Serum bilirubin at diagnosis	0.97 (0.90–1.04)	0.41
Age of diagnosis of UC or CD	0.98 (0.96–1.00)	0.14
Duration of UC or CD prior to PSC diagnosis	1.04 (1.01–1.07)	0.01
Age of diagnosis with PSC	1.01 (0.99–1.03)	0.51
Number of disease flares	1.13 (1.04–1.23)	0.006
UDCA use	1.36 (0.69–2.70)	0.38
Requirement for OLT	0.69 (0.40–1.21)	0.2
Mayo risk score	0.81 (0.67–1.00)	0.045
5-Aminosalicylate use	1.07 (0.61–1.88)	0.82
Azathioprine/6-mercaptopurine use	1.36 (0.56–3.27)	0.5
CD (relative to UC)	0.28 (0.10–0.73)	0.009

CI = confidence interval; UC = ulcerative colitis; CD = Crohn’s disease; PSC = primary sclerosing cholangitis; UDCA = ursodexoxycholic acid; OLT = orthotropic liver transplantation

**Table 6.** Cox proportional hazards analysis for colon dysplasia/cancer

Variable	Hazard ratio (95% CI)	*P*-value
Duration of UC or CD prior to PSC diagnosis (per 5 years)	1.22 (1.03–1.45)	0.021
Age of diagnosis with PSC (per 5 year)	1.07 (0.93–1.22)	0.36
Moderate/severe disease activity at diagnosis of IBD	5.28 (2.24–12.4)	<0.001
UDCA use	1.31 (0.62–2.78)	0.48
Requirement for OLT	1.09 (0.54–2.21)	0.80
Mayo PSC risk score = 1 (relative to 0)	0.82 (0.38–1.79)	0.62
5-Aminosalicylate use	0.91 (0.48–1.73)	0.78
Azathioprine/6-mercaptopurine use	1.26 (0.45–3.49)	0.66
CD (relative to UC)	0.44 (0.16–1.25)	0.12

CI = confidence interval; UC = ulcerative colitis; CD = Crohn’s disease; PSC = primary sclerosing cholangitis; UDCA = ursodexoxycholic acid; OLT = orthotropic liver transplantation

## Discussion

To our knowledge, the clinical outcomes of UC and CD patients with PSC have not been investigated and compared in a large cohort of patients from North America. Our study, representing the largest cohort of patients reported to date and comparing PSC-UC with PSC-CD followed in a tertiary care center, demonstrated that there was a trend for lower risk of colectomy and colon neoplasia in PSC-CD than in PSC-UC.

Patients with UC are at an increased risk of colorectal neoplasia when the duration of UC exceeds 10 years, with onset of the disease at a younger age and extensive involvement of the colonic mucosa [24]. Some studies have shown an increased risk of colon carcinoma in patients with CD [25–27], while some studies refute the possibility that CD is associated with an increased risk of colon carcinoma [28–31]; however in these studies where no increased risk of colon cancer was observed in CD patients; if duration of disease is taken into account, the risk of colon cancer is increased. Our study also observed that increasing duration of both UC and CD prior to PSC diagnosis was associated with increased risk of colon neoplasia.

The risk of colon cancer/dysplasia in the presence of PSC has also been investigated in both UC and CD. Although several studies have clearly demonstrated the increased risk of developing colitis-associated CRC in PSC-UC patients [7–12], it is unclear whether PSC also increases the risk of developing colon neoplasia in patients with colonic CD. The lack of evidence is probably due to UC being more commonly associated with PSC than is CD [1]. In a study from England, which compared CD patients with and without PSC, colonic CD did not seem to increase the risk for neoplasia of the colon [13]; however, in a study from Sweden, patients with PSC and CD were 6.7 times as likely than controls to develop colorectal dysplasia or cancer [4]. Also a large population-based study demonstrated that the risk of colon neoplasia in patients with CD was equal to that in patients with UC [5]; however, in this study cohort, only 5 of 78 patients with colon neoplasia had concurrent PSC [5]. In our patient cohort, we observed that the cumulative occurrence of colon cancer/dysplasia was more common in PSC-UC than in PSC-CD, with UC patients at 56% higher risk of developing colon neoplasia. Although the difference was not statistically significant on multivariate analysis, our study may have been underpowered to detect a significant difference. We did find a significant difference when the duration of disease was taken into account; PSC-UC patients had a higher risk of colon neoplasia than PSC-CD. We also observed that the clinical phenotype and presentation of CD in the presence of PSC is much different. CD behaved more like UC with extensive colitis in a majority of patients. Only three presented with fistulizing disease and none had peri-anal disease.

It was also interesting to observe the effect of PSC on the risk of colectomy. We had earlier shown that progressive PSC requiring OLT was associated with a reduced risk of colectomy in UC patients [14]. In the present study, including patients with UC and CD, we found that the requirement for OLT and a higher Mayo PSC risk score was associated with decreased need for colectomy, suggesting the inverse relationship between the severity of PSC as defined by the risk score and the requirement of OLT and the severity of IBD defined by colectomy. The possibility of lymphocyte trafficking in this clinical presentation remains to be explored.

We also observed that PSC patients in our cohort with concurrent UC had a 12% increased risk of colectomy over those with CD. Patients with UC had more flares, worse disease activity and higher risk of colon neoplasia, which may explain the higher risk of colectomy. As suggested earlier, although the difference was not statistically significant, our study may have been underpowered to detect a significant difference.

The severity of endoscopic inflammation increased the risk of colon neoplasia, similarly to previous studies in which endoscopic and histological inflammation correlated with the risk of neoplasia [32, 33].

Our study is clinically significant for a number of reasons. We found that PSC patients with concurrent UC had more flares, worse disease activity, and a trend towards higher risk of colon neoplasia and also colectomy, suggesting a progression risk that is biologically different from that of PSC-CD. Although the difference is not significant, it may be clinically relevant and our study was probably underpowered to detect a difference; however, it was also interesting to note that CD in PSC patients presents with colonic disease with occasional small bowel involvement and, in most cases, mimics UC. The other limitation is that PSC patients in our cohort had a high risk of colectomy and colon neoplasia compared with previous reports, probably because of tertiary referral center bias.

There are certain limitations to our study. The study population was recruited from a subspecialty tertiary care referral center. This contributed to a referral bias. Although there was a trend towards worse disease in patients with concurrent UC, the study may have been underpowered to find a significant difference. We excluded patients with indeterminate colitis because of low numbers; this was probably because of long follow-up, a number of patients initially diagnosed with indeterminate colitis were re-diagnosed with either UC or CD. Nevertheless, this is the largest cohort study on the natural history of PSC and concurrent UC or CD.

To conclude, PSC-CD appears to be associated with a lower risk of colon neoplasia and colectomy than PSC-UC and has a milder clinical course.

**Disclaimer:** part of the paper was presented as an oral presentation at the Digestive Disease Week 2012, San Diego, CA, USA

*Conflict of interest statement:* none declared.
